# A cell culture platform for *Cryptosporidium* that enables long-term cultivation and new tools for the systematic investigation of its biology

**DOI:** 10.1016/j.ijpara.2017.10.001

**Published:** 2018-03

**Authors:** Christopher N. Miller, Lyne Jossé, Ian Brown, Ben Blakeman, Jane Povey, Lyto Yiangou, Mark Price, Jindrich Cinatl, Wei-Feng Xue, Martin Michaelis, Anastasios D. Tsaousis

**Affiliations:** a Laboratory of Molecular & Evolutionary Parasitology, RAPID Group, School of Biosciences, University of Kent, Canterbury, UK; b School of Biosciences, University of Kent, Canterbury, UK; c Industrial Biotechnology Centre, School of Biosciences, University of Kent, Canterbury, UK; d School of Physical Sciences, University of Kent, Canterbury, UK; e Institut für Medizinische Virologie, Klinikum der Goethe-Universität, Frankfurt am Main, Germany

**Keywords:** *Cryptosporidium*, Cell culture, COLO-680N, Lipidomics, Proteomics, Atomic force microscopy, Immunofluorescence microscopy, Electron microscopy

## Abstract

*Cryptosporidium* parasites are a major cause of diarrhoea that pose a particular threat to children in developing areas and immunocompromised individuals. Curative therapies and vaccines are lacking, mainly due to lack of a long-term culturing system of this parasite. Here, we show that COLO-680N cells infected with two different *Cryptosporidium parvum* strains produce sufficient infectious oocysts to infect subsequent cultures, showing a substantial fold increase in production, depending on the experiment, over the most optimistic HCT-8 models. Oocyst identity was confirmed using a variety of microscopic- and molecular-based methods. This culturing system will accelerate research on *Cryptosporidium* and the development of anti-*Cryptosporidium* drugs.

## Highlights

• We have developed a cultivation system for *Cryptosporidium* that enables long-term culturing.
• Lipidomics fingerprinting was used to demonstrate the identity of the oocysts.
• Atomic force microscopy imaging was performed for thin- and thick-walled *Cryptosporidium* oocysts.
• The cell culturing is easy to handle and enables propagation and cryopreservation.
• The new tools can be used for a detailed investigation of *Cryptosporidium*’s biology.

## Graphical abstract

**Figure:**
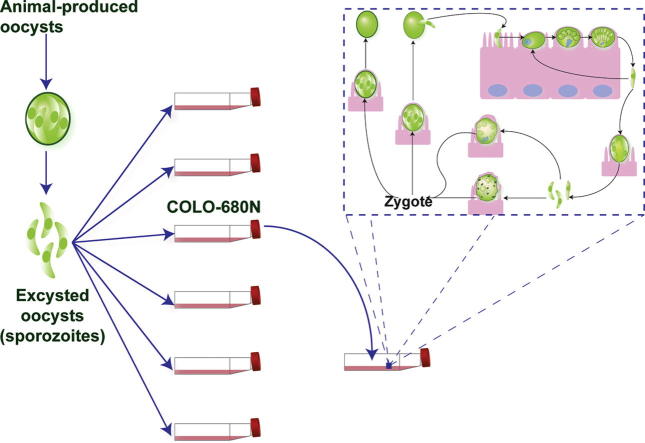

Cryptosporidiosis causes a significant number of deaths in children and immunocompromised individuals (Kotloff et al., 2013). It is caused by species of the genus *Cryptosporidium*, in humans typically by *Cryptosporidium parvum* and *Cryptosporidium hominis*. The *Cryptosporidium* spp. belong to the phylum Apicomplexa and it has recently been proposed for the species to be reclassified as a member of the subclass of gregarine (Ryan et al., 2016). They are waterborne pathogens, and cryptosporidiosis has commonly been associated with disease in developing countries. However, more recent molecular epidemiological studies suggested that the disease is also an increasing health concern in developed countries and may have reached epidemic levels (Kotloff et al., 2013, Checkley et al., 2015). Only one moderately effective drug (nitazoxanide) is available for the treatment of cryptosporidiosis. More effective drugs are urgently needed (Checkley et al., 2015).

*Cryptosporidium* is a parasite that invades host cells, within the boundaries of the host cell membrane, residing intracellularly yet extra-cytoplasmic, sometimes referred to simply as epicellular (Ryan et al., 2016). *Cryptosporidium* typically infects epithelial tissues of the upper intestinal tract, accompanied by localised deterioration of microvilli. In immunocompromised individuals, the parasite can also be found in other epithelial tissues including most of the upper stages of the digestive and respiratory tracts as well as other unrelated organ systems (Sponseller et al., 2014). The *Cryptosporidium* life cycle is complex and involves a number of intracellular/extracytoplasmic and extracellular stages, resulting in oocysts that contain the infective sporozoites (Supplementary Fig. S1).

A cell culture system that enables continuous *Cryptosporidium* cultivation and systematic elucidation of the *Cryptosporidium* life cycle, especially the endogenous phases, is missing. Previous approaches have been hampered by problems including rapid senescence of primary cell cultures, incomplete parasite life cycles, and insufficient production of sporulated infective oocysts (Karanis and Aldeyarbi, 2011, Checkley et al., 2015). The current methods used to produce infective *Cryptosporidium* oocysts, aside from small-scale cultures in vitro, require continuous infection of animals, typically neonatal cows or sheep and sometimes mice (Vinayak et al., 2015). Due to a lack of cryopreservation methods, oocysts cannot be stored and need to be freshly prepared on a continuous basis. A recent publication tackled the challenge of cell culture-based oocyst production using a hollow fiber technology that mimics the gut (Morada et al., 2016). However, specialised equipment is needed and the required cell culture media supplements are expensive. In addition, the system does not enable study of the *Cryptosporidium* life cycle and biology in real time at a cellular level in the context of a host cell.

Here, we show that inoculation of COLO-680N cultures with *C. parvum* produced sufficient amounts of infective oocysts to enable sustainable propagation of the parasite in standard tissue culture at a laboratory scale. We tested a panel of seven human cancer cell lines (using methods described in Supplementary Data S1) for their capacity to support *C. parvum* propagation including COLO-680N (oesophageal squamous-cell carcinoma), DLD-1 (colon adenocarcinoma), KYSE-30 (oesophageal squamous-cell carcinoma), HCT-15 (colorectal adenocarcinoma), SJSA-1 (osteosarcoma), MKN-1 (gastric carcinoma), and the colon adenoma carcinoma cell line HCT-8, which has most commonly been used for the investigation of *Cryptosporidium* in cell culture (Hijjawi et al., 2001). However, *Cryptosporidium*-infected HCT-8 cultures do not produce enough infective oocysts to maintain infected cultures (Muller and Hemphill, 2013), which also raises concerns about the suitability of HCT-8 for the study of *Cryptosporidium* biology. The cell lines were infected with the *C. parvum* strain Moredun (Girouard et al., 2006) using a total input of 5 × 10^5^ of excysted oocysts per 10 mL of medium (25 cm^2^ flask). After an incubation period of 2 weeks, COLO-680N cultures were the only ones that had produced substantially more oocysts (approximately 40-fold higher) than the number of input oocysts (Fig. 1A, Supplementary Table S1). While HCT-8 cells died after a few days of infection, COLO-680N cultures remained viable and produced oocysts for almost 8 weeks without sub-culturing, requiring only weekly medium exchange (Fig. 1B). As a result, total *Cryptosporidium* oocyst production in the COLO-680N cell line (number of oocysts produced) exceeded the HCT-8-mediated oocyst production (2.5 × 10^5^ oocysts/mL of culture medium) by 20 times (5 × 10^6^) after 10 days of incubation (Fig. 1C). At day 60, COLO-680N cells had produced an accumulated number of 1.2 × 10^7^ oocysts/mL of culture medium obtained from weekly harvests. Given that the initial oocyst count was 1 × 10^5^ oocysts/ml, this represents a 50-fold increase in oocyst numbers at 10 days p.i. and a 120-fold increase by the end of the culture. Also of note, oocysts derived from the supernatants of COLO-680N cell cultures, but not from the supernatants of HCT-8 cell cultures, enabled the infection of novel cell cultures (Supplementary Fig. S2C). Infection of COLO-680N cells with cattle-derived *C. parvum* oocysts resulted in similar amounts of infective oocysts in 25 independent experiments. In addition, we performed three rounds of infection using COLO-680N culture-derived oocysts without noticing changes in oocyst production efficacy, showing that COLO-680N cells are suited for the continuous long-term cultivation of *C. parvum* oocysts. Continuous *C. parvum* infections of COLO-680N cells were confirmed using PCR primers specific to *C. parvum* DNA, which displayed the presence of parasite DNA in both the cell monolayer (Fig. 1D), and media fractions of the two-dimensional (2-D) cultures (Fig. 1E). *Cryptosporidium-*specific primers did not produce bands in non-infected COLO-680N cells (Fig. 1D and E; Supplementary Fig. S3). The amplified DNA regions were sequenced to confirm their identity. In addition, purified COLO-680N-produced oocysts were visualised by scanning electron microscopy (Fig. 1F). To evaluate our results even further, we repeated the infection experiments using freshly excysted and purified sporozoites; the purity of the sporozoites (absence of oocysts in the sample) was validated using bright-field microscopy. Quantitative PCR (qPCR) has demonstrated the production of 2.4 × 10^6^ oocysts (from an initial inoculation of 1 × 10^6^ sporozoites, Fig. 1I), 9 days p.i., and fluorescence microscopy confirmed the presence of newly produced oocysts in the medium (Fig. 2E). The discrepancy in the numbers of oocysts produced (from the other experiments) could be a result of the oocyst treatment/purification, the detection method using qPCR (Shahiduzzaman et al., 2009), or the presence of a high amount of host cell material (debris and RNA) that could inhibit the reaction. The produced oocysts were used in two rounds of infection. The first round of infection was done in triplicates in 12 × 25 cm^2^ flasks format and the presence of oocysts was assessed by standard PCR analysis using Heat shock protein 70 (Hsp70)-specific primers (Supplementary Table S2). Then crudely purified oocysts were used to re-infect fresh COLO-680N cells, and the production of fresh ones was further evaluated (Supplementary Fig. S4).

**Fig. 1:**
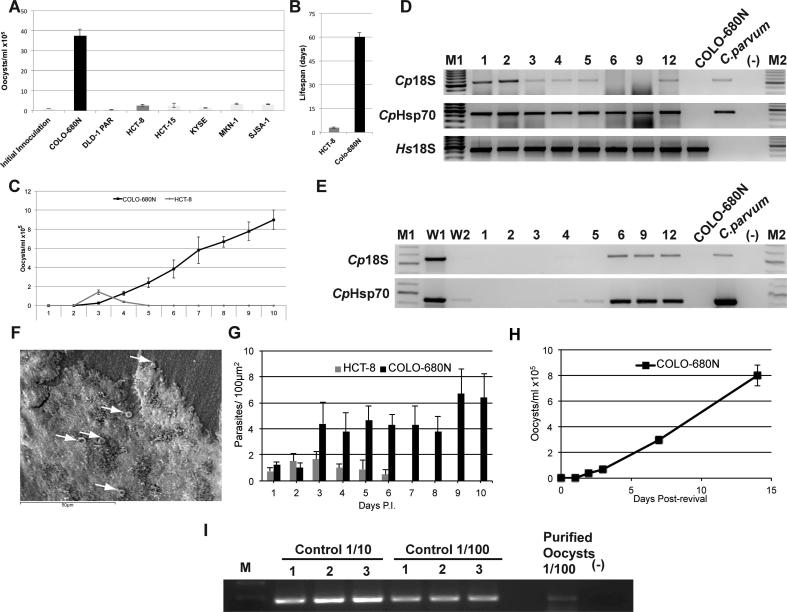
Cell culture-based production of C*ryptosporidium* parvum oocysts (A) A bar chart representing the average *C. parvum* oocyst production (mean ± S.D. from three independent experiments) in the investigated cell lines after initial infection with 1 × 10^5^ excysted oocysts. Final oocyst counts are representative of total content recovered after 14 days of incubation, regardless of host cell viability. Oocysts were recovered from cell culture media via saturated salt-column chromatography and counted via haemocytometer. Initial experiments, infecting excysted oocysts, returned a near 40-fold return in oocysts by COLO-680N cultures, compared with only a two-fold return by HCT-8 cells. (B) Bar chart of the time span during which oocysts were produced by COLO-680N and HCT-8 cultures after a single initial inoculation, representing the time from when the first oocysts were detected in the harvest media until the last time an oocyst was detected (mean ± S.D. from three independent experiments). (C) *Cryptosporidium parvum* oocyst production in COLO-680N and HCT-8 cancer cells over a 10 day period after an inoculation with 1 × 10^5^ excysted oocysts, measured through daily sampling via the same means as (A) (mean ± S.D. from three independent experiments). (D) PCR amplification of *C. parvum* 18S RNA (*Cp*18S, 580 bp, primers CF/CR) and heat shock protein 70 (Hsp70) (*Cp*Hsp70, 462 bp, primers Hsp70F4/Hsp70R4) DNA fragments from *C. parvum*-infected COLO-608N cells. A *Homo sapiens* 18S DNA fragment (*Hs*18S, 418 bp, primers *Hs*18S1F/*Hs*18S1R) demonstrates abundance of host cell DNA in the sample. DNA extraction was performed at days 1, 2, 3, 4, 5, 6, 9 and 12 p.i., from DNA extracted from cells removed from culture flasks via trypsin and washed multiple times at low speed (300 *g*) to remove extracellular stages. Cattle-derived *C. parvum* oocysts (*C. parvum*) and uninfected COLO-680N cells (COLO-680N) served as controls. M1 is the 1 kb DNA ladder from Promega (UK). M2 is the 100 bp DNA ladder from Promega. (E) PCR amplification of *C. parvum* 18S RNA (*Cp*18S, 580 bp, primers CF/CR) and Hsp70 (*Cp*Hsp70, 462 bp, primers Hsp70F4/Hsp70R4) DNA fragments from samples derived from the supernatants of *C. parvum-*infected COLO-608N cells via percoll gradient after excystation. Input oocysts were removed by two washing steps with PBS (W1 and W2) 6 h p.i., leaving no detectable *C. parvum* DNA in suspension. Time points and controls were the same as described in (D). (F) Scanning electron microscopy of COLO-680N produced *C. parvum* oocysts. White arrows indicate *Cryptosporidium* oocysts. (G) Bar chart demonstrating the average number of *C. parvum* infections within cells in a 100 μm^2^ oil field at 1000x magnification at days 1 to 10 p.i., This data represents absolute infection numbers only as multiple infections per cell were possible (mean ± S.D. from five independent experiments). Parasites were identified as the presence of co-localised propidium iodide and Sporo-glo within a host cell. (H) Oocyst production in *C. parvum-*infected COLO-680N cell cultures after 2 weeks of cryopreservation and resuscitation (mean ± S.D. from three independent experiments). (I) Agarose gel analysis of a quantitative PCR (qPCR) experiment with *C. parvum*-specific Hsp70LJ primers. Cattle-produced *C. parvum* oocysts were used as controls in two different DNA dilutions (1/10 and 1/100) of an initial concentration of 2 × 10^5^ oocysts. qPCR was performed at day 9 (purified oocysts 1/100), where it amplified a corresponding band to estimate the analogous concentration of oocysts. M is the 1 kb DNA ladder from Promega.

**Fig. 2:**
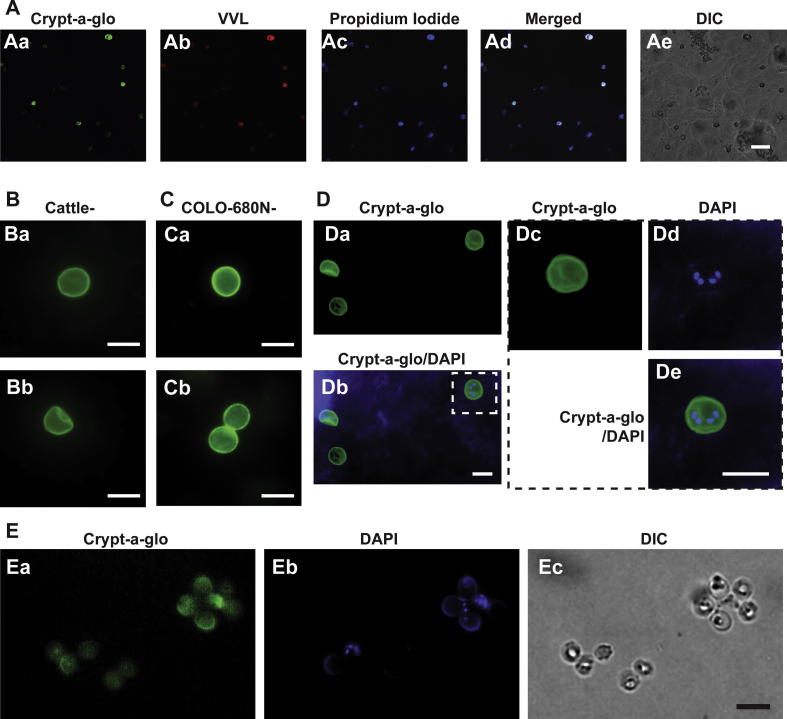
*Detection of* C*ryptosporidium* parvum *using different specific staining methods.* All images were acquired 6 days p.i., after fixation and permeabilisation. (A) Visualisation of *C. parvum* oocysts in infected COLO-680N cells. *Cryptosporidium parvum* oocysts were detected using Sporo-glo (Waterborne, USA), a fluorescein-labelled mouse monoclonal antibody binding to *Cryptosporidium* (Aa), CpClec, that binds to the surface of the apical region and to dense granules of sporozoites and merozoites (Bhalchandra et al., 2013) (Ab), and DAPI staining (Ac) that can be used to distinguish between host cell nuclei and parasites by morphological inference when coupled with differential interference contrast (DIC) and other stains. (Ad) Merge of (Aa-c) conclusively showing that what is being observed is indeed *C. parvum* oocysts. (Ae) The corresponding DIC microscopy image. Scale bar = 40 μm. VVL, *Vicia villosa* lectin*.* (B) Crypt-a-glo-stained cattle-produced oocyst. Scale bar = 5 μm. (C) Crypt-a-glo-stained COLO-680N-produced oocysts. Scale bar = 5 μm. (D) COLO-680N-produced oocysts stained with Crypt-a-glo and DAPI. (Da). Crypt-a-glo, (Db) DAPI-merge). (Dc-e) Inset from Db showing an oocyst at higher magnification, indicating DAPI staining of the four sporozoites. Scale bar = 5 μm. (E) COLO-680N-produced oocysts from a culture infected only with purified sporozoites stained with Crypt-a-glo and DAPI. (Ea) Crypt-a-glo, (Eb) DAPI, (Ec) DIC. Scale bar = 10 μm.

The identity of the COLO-680N-produced *C. parvum* oocysts was further confirmed using different specific staining methods. Crypt-a-glo (Waterborne™; an antibody that recognises the oocyst cell wall), *Vicia villosa* lectin (VVL, Vector laboratories, UK); binds to O-glycan mucin repeats on *C. parvum* sporozoites), a mucin-like glycoprotein that contains a C-type lectin domain (CpClec; binds to surface of the apical region and to dense granules of sporozoites and merozoites (Bhalchandra et al., 2013)) and direct sporozoite staining using propidium iodide and Sporo-glo (Waterborne™) resulted in virtually identical staining patterns in *C. parvum*-infected COLO-680N cells, indicating the presence of oocysts and other non-extracellular life stages of *Cryptosporidium* (Fig. 2A; Supplementary Figs. S5–S7; Supplementary Movie S1). Crypt-a-glo staining did not reveal any significant differences between COLO-680N- and cattle-produced oocysts (Fig. 2B and C). Closer examination of the produced oocysts did, however, appear to demonstrate two morphological populations, which has been observed in *C. parvum* cultures previously (Thompson et al., 2005) (Fig. 2D; Supplementary Movie S1). The comparison of Crypt-a-glo staining of *C. parvum*-infected COLO680N- with HCT-8 cells further confirmed that *C. parvum-*infected COLO-680N cultures are characterised by enhanced numbers of infected cells compared with *C. parvum*-infected HCT-8 cultures (Fig. 1G; Supplementary Fig. S5). To finally confirm the production of fresh oocysts, Crypt-a-glo stained oocysts were excysted (Supplementary Fig. S2A) and used for the infection of COLO-680N cultures. Then, cell cultures were washed to remove remaining Crypt-a-glo stained oocysts. Upon harvesting, neither the infected cultures nor the newly produced oocysts displayed Crypt-a-glo staining. However, oocysts were detected using DAPI, indicating that indeed new oocysts were produced (Supplementary Fig. S2). We also subsequently have been able to propagate successfully the alternative *C. parvum* Iowa strain in COLO-680N cells (Supplementary Fig. S7).

In addition, we have attempted to resolve the issue of lacking of a cryopreservation system that enables the long-term storage of infective *Cryptosporidium* parasites. Here, *C. parvum* strain Moredun-infected COLO-680N cells were cryopreserved, stored for 2 weeks at −80 °C, and resuscitated by standard protocols used for cell cultures. Three days after resuscitation, the cultures started to produce oocysts similar to freshly infected COLO-680N cultures (Fig. 1H). This demonstrates that *C. parvum*-infected COLO-680N can be cryo-conserved, providing the first known long-term storage system for *Cryptosporidium*.

Next, we compared *C. parvum*- and non-infected cell cultures by a MALDI-MS-based fingerprinting approach. Principal Component Analysis (PCA) of the pre-processed data, as described in Supplementary Data S1 and in more detail in Povey et al. (2014), resulted in separate groupings of the COLO-680N, but not the HCT-8 samples (Supplementary Fig. S8A). We found substantial alterations in the fingerprints between non-infected and *C. parvum*-infected COLO-680N cells 5 days after infection, but not between non-infected and *C. parvum*-infected HCT-8 cells (Supplementary Fig. S8B–E). These findings suggest *C. parvum* infection resulted in a more noteworthy difference in COLO-680N cultures compared with HCT-8, suggesting either a more successful infection (the presence of an increased number of *Cryptosporidium*-originated proteins) or a more pronounced effect on the host cell proteome during infection.

Furthermore, we compared COLO-680N- and cattle-produced *C. parvum* oocysts by a lipidomics approach and by atomic force microscopy (AFM). The lipidomics characterisation was performed using MALDI-TOF MS for the analysis of lipids within the range of 600 to 2,000 Da (Supplementary Fig. S9A–D). Graphical representation of the Principal Components (PC1 and PC2) from PCA (Povey et al., 2014) showed groupings which could not substantially differentiate between the oocysts of commercial (Bovine) or laboratory (COLO-680N) origins (Supplementary Fig. S9E and F). To investigate the existence of oocysts at the highest magnification possible, we employed AFM that has been used previously to elucidate unique surface details at a level of resolution not visible using any other imaging modalities in other parasites (e.g. *Giardia* and *Trypanosoma* (Dvorak et al., 2000)). Notably, we observed two types of oocysts by AFM in *C. parvum*-infected COLO-680N cultures (Supplementary Fig. S10C and D). We found a larger type of COLO-680N-produced oocyst (Supplementary Fig. S10) that was indistinguishable from cattle-produced oocysts by force-distance curve-based imaging (Supplementary Fig. S10A and B; Supplementary Movie S2). These oocysts are likely to represent traditional, thick-walled oocysts, since they are the larger and more rigid of the structures and more closely resemble those produced by the cattle (Thompson et al., 2005). We also identified a smaller type of oocyst that upon casual observation appeared less structurally rigid and may represent the thin-walled oocysts (Supplementary Fig. S10D).

In summary, we present a cell culture system that enables the sustainable, continuous propagation of infective *C. parvum* oocysts and the systematic investigation of *Cryptosporidium* oocysts. Previously, attempts to cultivate *Cryptosporidium* in cell culture were affected by a lack of production of sufficient amounts of infective *C. parvum* oocysts (Tzipori and Widmer, 2008, Checkley et al., 2015) or required sophisticated, expensive specialist equipment and methodologies to support 3-D cultures that are not commonly available to research laboratories (Morada et al., 2016). Moreover, 3-D cultures do not enable study of the *C. parvum* biology (Morada et al., 2016). In contrast, COLO-680N cells enable *C. parvum* propagation, the sustainable production of infective *C. parvum* oocysts, and the investigation of *C. parvum* biology at a laboratory scale in standard tissue cultures with commonly available equipment and knowledge. In addition, these data demonstrate a long-term maintenance of the cell line and subsequently a prolonged production of oocysts. The reasons behind this observation are unknown, but previous studies on COLO-680N have suggested that the expression of high levels of fatty acid synthase might promote cell viability (Orita et al., 2010). This could be beneficial to the parasite that depends on host cell lipid synthesis, since it is unable to synthesize fatty acids de novo (Zhu, 2004).

In conclusion, the discovery of COLO-680N as a cell culture platform for the production of *C. parvum* will provide a step-change with regard to research on *Cryptosporidium* as follows: (i) it is the first easy-to-handle system that enables the long-term sustainable production of infective oocysts at a laboratory scale and removes the constant dependence on immunosuppressed animals for production of *Cryptosporidium* oocysts along with all its ethical implications; (ii) *C. parvum*-infected cell cultures can be frozen and stored. Prior to the establishment of the COLO-680N cultivation system for *C. parvum*, oocysts had to be freshly acquired from animals and could not be stored over longer periods; (iii) our study paves the way for establishment of compound-screening platforms for the identification of anti-*Cryptosporidium* drugs and the systematic elucidation of *Cryptosporidium* biology, including the utilisation of a CRISPR transfection system for *Cryptosporidium* (Vinayak et al., 2015). Hence, the COLO-680N-based platform for *C. parvum* propagation will enable a much larger community to work on *Cryptosporidium* and open unprecedented opportunities to decipher *Cryptosporidium* biology and to develop anti-*Cryptosporidium* therapies.

## Appendix A Supplementary data

Supplementary Table S2

Supplementary Movie S1 *Cryptosporidium parvum* oocyst captured by Atomic Force Microscopy (AFM). Three-dimensional topology animation displaying the overall morphology of the COLO-680N-produced oocyst displayed in Supplementary Fig. S10A.

Supplementary Movie S2 Animation of cellular staining of *Cryptosporidium* using confocal microscopy. Three-dimensional (3-D)-stacked animation displaying the infection of COLO-680N cells with *Cryptosporidium parvum* (stained with Crypt-a-glo; green) displayed in Supplementary Fig. S6.
