# Supplementary material for: A cell culture platform for Cryptosporidium that enables long-term cultivation and new tools for the systematic investigation of its biology
Source: Int J Parasitol. 2018 Mar;48(3-4):197–201. doi: 10.1016/j.ijpara.2017.10.001 (PMC5854368; doi:10.1016/j.ijpara.2017.10.001)
Supplement: Supplementary Data S1 [file mmc1.docx]

1. **Supplementary methods**

## Cell culture

The cell lines used in this study were: COLO-680N (human oesophageal squamous-cell carcinoma), obtained from CLS Cell Line Services, Eppelheim, Germany; DLD-1 (human colon adenocarcinoma), KYSE-30 (human oesophageal squamous-cell carcinoma) and HCT-15 (human colorectal adenocarcinoma), obtained from Deutsche Sammlung von Mikroorganismen und Zellkulturen GmbH (DSMZ), Braunschweig, Germany; SJSA-1 (osteosarcoma) and HCT-8 (ileocecal colorectal adenocarcinoma), obtained from American Type Culture Collection (ATCC), Manassas, VA, US, Cat Nos. CRL-2098 and CCL-244, respectively; MKN-1 (gastric carcinoma), obtained from Japanese Collection of Research Bioresources (JCRB) Cell Bank, Osaka, Japan (Supplementary Table S1).

Cells were cultivated in RPMI-1640 medium (Sigma-Aldrich, UK, Cat No R8758) supplemented with 10% FBS (Sigma-Aldrich, Cat No F8084), 100 U/mL of penicillin, 100 µg/mL of streptomycin and 250 ng/mL of amphotericin B (Antibiotic Antimycotic solution, Sigma-Aldrich, Cat No A5955) at 37 °C and 5% CO_2_.

- 1. *Excystation of Cryptosporidium parvum oocysts and infection of cell lines*

*Cryptosporidium parvum* oocysts were provided by the Creative Science Company, pre-purified and identified as the ‘Moredun’ strain, which was originally acquired from the gastrointestinal contents of a dead red deer calf (*Cervus elaphus*), sourced from the Glen Saugh experimental deer farm (Scotland, UK) in 1987 (Girouard et al., 2006; Bouzid et al., 2013). *Cryptosporidium parvum* oocysts (Iowa strain) were obtained from Bunch Grass Farm (Idaho, USA). For a typical infection, 1 x 10^5^ oocysts, counted prior to excystation, were used to infect T25 cell culture flasks at between 70-80% confluency (1.7 to 2 x 10^6^ cells) giving a multiplicity of infection (MOI) of approximately 0.5. All infections were conducted in triplicate for reliability.

Excystation was achieved by adding 100 μL of 0.01% Trypsin and 400 μL of 0.5% Sodium Hypochlorite to a pellet containing the desired number of oocysts (Upton et al., 1994; Feng et al., 2006). The homogenised mixture was then incubated in a 37^o^C water bath for 1 h, with intermittent vortexing. The excystation procedure was monitored by phase contrast microscopy, using a haemocytometer and stopped when visible sporozoites exceeded 80% of the theoretical maximum (4x the original number of oocysts present per sample). Samples were then pelleted at 2,200 *g* for 8 min, and suspended in cell culture medium prior to cell infection. Approximately 24-26 h p.i., T25 flasks were washed twice with 10 ml of 1x PBS, to remove un-excysted oocysts and remaining sporozoites. Fresh medium then added. In order to assess the effectiveness of the p.i. washes, each wash was subjected to PCR testing (see Section 1.6) for the presence of *C. parvum* DNA. A negative result would confirm that the only remaining *C. parvum* at this point would be intracellular stages, i.e. successful infections. Therefore any future positive PCR results would indicate the presence of a successful infection, *Cryptosporidium* extracellular stages and production of oocysts after the initial infection (see Fig. 1). Enumeration of oocysts produced by the cultures was achieved via phase contrast microscopy of the purified oocyst fractions, using a standard haemocytometer. Where appropriate, estimates of infection intensity were also made using a fluorescence approach, where the number of *C. parvum* stages in a 1,000 x magnified field of view, identified by Sporo-glo or Crypt-a-glo, were averaged from 10 randomly selected areas of a stained culture. This method was used primarily in assessing the success of an infection for quality control purposes.

## Purification of Cryptosporidium from infected cultures

Growth media from infected cultures and a subsequent 5 mL wash (with 1x PBS) were collected. The suspensions were centrifuged at 500 *g* for 5 min to remove host cells and debris. The supernatant, which contained the oocysts, was then transferred to fresh tubes, and the oocysts were pelleted by centrifugation at 2,100 *g* for 8 min. The pellets were re-suspended in 1 mL of 1x PBS and carefully laid on top of 9 mL of saturated (37%) sodium chloride solution, in a 15 mL Falcon tube. These two layers were then topped up with 1 mL of sterile water before being centrifuged at 2,100 *g* for 8 min. This centrifugation step resulted in the formation of a milky white phase between the PBS and salt layers, which contained the live oocysts. Using this methodology, empty and non-viable oocysts fall to the bottom of the tube during centrifugation (Chen and Huang, 2006). The oocysts were carefully pipetted from the interface. Because the isolated oocysts may contain a carryover from the sodium chloride solution, 9 mL of 1x PBS were added to dilute the mixture and the oocysts were pelleted again at 2,100 *g* for 8 min. The pipetting/dilution steps were repeated twice.

- 1. *DNA extraction from oocysts and infected cultures*

COLO-680N cells, infected using the above protocol, were washed twice with 1x PBS prior to DNA extraction. Samples were taken 6 h p. i. and subsequently on days 1, 2, 3, 4, 5, 6, 9 and 12.

For COLO-680N cells and intracellular but extra-cytoplasmic *Cryptosporidium* stages, DNA extraction was performed using the Qiagen DNeasy Blood and Tissue kit (Qiagen, UK, Cat. No 69504) following the manufacturer's instructions. For *C. parvum* oocysts, samples were collected from cell culture supernatants as described in Section 1.3. Then, DNA extraction was performed using the Omega (UK) E.Z.N.A. fungal extraction kit (Cat. No. D3390-1) following the manufacturer's instructions. Extracted DNA was quantified using a NanoDrop 1000 Spectrophotometer.

- 1. *Excystation, purification and infection with C. parvum sporozoites*

*Cryptosporidium parvum* oocysts were excystated following the routine procedure described in Section 1.2. Sporozoites were additionally filtered through a 3.0 μm filter to remove any residual oocysts, which may have been resistant to the treatment. The purity (lack of oocysts in the sample) of the oocysts was monitored by observing the sample under 600 magnified field microscopy prior and after the excystation and purification procedures. After purification, we were not able to observed any oocysts in the samples. COLO-680N host cells were then infected and following 24 h, medium was replaced by fresh medium as usual. Nine days p.i., the oocysts suspension was separated from the infected cells following the procedure described below.

The supernatant/ cell culture medium was crudely cleared by centrifugation at 800 *g* for 1 min (to remove suspended COLO- 68ON cells), followed by further clearing through a 10 μM filter. This procedure, however, does not fully remove cell debris. The oocysts were then analysed by microscopy and quantitative PCR (qPCR) analysis using qPCRBIO SyGreen Mix Lo-ROX from PCR BIOSYSTEMS (UK) (see Section 1.6).

- 1. *PCR amplification*

The PCR contained the following reagents in a 50 μL total volume: 10 μL of PCR 5x Flexibuffer, 2 μL of MgCl_2_, 1 μL of dNTPs (10 mM), 2 μL of forward primer (10 mM), 2 μL of reverse primer (10 mM), 31.75 μL of H_2_O, 0.25 μL of GoTaq G2 Hot Start Polymerase (Promega, UK, Cat. No. M740A) and 1 μL of the extracted DNA. The list of primers used in the different reactions is shown in Supplementary Table S2.

PCR set-up conditions were as follows: one cycle of 95 ^o^C of initial denaturation for 5 min, followed by 36 cycles of 35 s denaturation steps at 95 ^o^C, 20 s annealing steps at 52 ^o^C, 20 s elongation steps at 72 ^o^C and a final 10 min elongation step at 72^o^C.

The resulting amplified DNA was visualised on a 1.8% agarose-Tris-acetate-EDTA (TAE) gel, stained with ethidium bromide.

- 1. *qPCR*

For qPCR analysis, we followed standard assay procedures and these were in accordance with those described in the The Minimum Information for Publication of Quantitative Real-Time PCR Experiments (MIQE) guidelines (Bustin et al. 2011). Genomic material was analysed between 1-3 days of harvesting oocysts. On harvesting, the oocyst suspension was pelleted and washed once with 1x PBS prior to filtering through a Pluriselect (UK) 10 mM filter. For a positive control, oocysts were taken from our *C. parvum* stock (2 x 10^7^ oocysts per ml), reference Iowa strain. All samples were stored at 4°C. For amplification, we used the 2 x qPCR SyGreenx-Lo-ROX from PCR Biosystems, as described by the supplier. This reagent is compatible with the BIORAD (UK) Chromo4 System. The cycler supports the Opticon Monitor Version 3.1. We used SB012 primers (Supplementary Table S2), previously described by Wu et al. (2000). These primers target cgd4_2390, which shares similarity with the vacuolar VPS35 protein. The gene reference is XM_625827.1.

The limit of detection of the SyGreen reagent is not stated by the supplier. The analytical specificity was determined by analysing the primer melting curve. A unique sharp peak at 60 °C indicated that annealing was specific and that only one PCR product was amplified. This was nonetheless verified by running a fraction of the PCR on an agarose gel.

Analysis in triplicate of qPCR profiles (log scale) and gel bands revealed that accurate numbers were within 1/10 to 1/1000 dilution (equivalent to 200- 20,000 oocysts). For numbers below 200, the signal to noise ratio was no greater than the negative control (no oocysts), making quantitation unreliable. Finally, while there was little variation between control samples (reference Iowa strain), variability was much greater among produced oocysts. It is likely that cell debris interfered with PCR efficiency.

- 1. *Electron microscopy (EM)*

UV sterilised 0.5 cm diameter 200 μm thick aclar film discs (Honeywell international Inc., India, 5042525) were deposited into a 24-well plate. For each well, 1 mL of COLO-680N cells at a concentration of 2.4 x 10^4^ cells/ml was added. Once the cells had reached 50 to 60% confluency, the cultures were infected with 50,000 *C. parvum* oocysts, giving an approximate MOI of 0.4. On days 6, 7, 8, 9 and 12, the supernatant was removed and cells were washed with 200 mM cacodylate buffer (pH 7.4). The buffer was aspirated and 1 mL of fixative, containing 2.5% Glutaraldehyde in 100 mM cacodylate buffer (pH 7.4), was then added and left at 4 ^o^C overnight. Next, each well was washed twice (10 min) with cacodylate buffer prior to staining with 1 mL of 1% osmium for 30 min at room temperature. The samples were washed and dehydrated through an ethanol series (30%, 50%, 70%, 90% and twice at 100%) before being embedded in Agar (Agar Scientific, UK) low viscosity resin.

Sections were cut initially with a glass knife and ultrathin sections of 65 nm were cut with a Diatome diamond knife on a RMC-MTXL ultramicrotome. The sections were placed onto 400 mesh uncoated copper grids. The grids were then stained for 45 min in 4.5% aqueous Uranyl Acetate, washed again, and subsequently stained for 7 min with Reynold’s lead citrate. Stained grids were then dried for 10 min before being visualised with a Jeol 1230 Transmission Electron Microscope at 80kV equipped with a Gatan Multiscan digital camera.

## Fluorescence microscopy

Cultures of COLO-680N and HCT-8 cells at 50-60% confluency were infected with *C. parvum* oocysts in 2-well permanox base chamber slides (Sigma-Aldrich, Cat No C6682), with an approximate MOI of 0.4. At harvesting points, cultures were washed with 1x PBS and then fixed in methanol for 10 min at room temperature. Next, the methanol was removed and the cells were permeabilised with 0.002% Triton-X100 in 1x PBS at room temperature for 30 min. Cells were then washed three times prior to incubation for 1 h with *Cryptosporidium-*specific antibodies (Crypt-a-glo, Waterborne^TM^, USA, dilution 1:10; Sporo-glo, Waterborne^TM^, dilution 1:10; anti-CpClec antibody (Bhalchandra et al. 2013), dilution 1:60), propidium iodide (500 nM), or *Vicia villosa* lectin (Alcantara Warren et al. 2008; Sharling et al. 2010) (VVL, 0.5 μg/ml).

Cells were washed a further three times with 1x PBS and then mounted with an aqueous mounting medium. This was done using either Fluromount (Sigma-Aldrich, Cat No F4680, with no DAPI) or Fluoroshield (Sigma-Aldrich, Cat No F6057, with DAPI). Slides were visualised by fluorescence microscopy using an Olympus 1X82 or Zeiss Elyra P1 confocal microscope.

## Atomic force microscopy (AFM)

Oocysts (1 x 10^5^) were suspended in 25 μL of 1x PBS, pipetted onto a freshly cleaved mica sheet pre-mounted on a magnetic specimen disc (Agar Scientific) and left to sediment for 1 h at 4^o^C in a humidified chamber. Oocysts were then fixed for 1 h with 25 μL of 5% Glutaraldehyde in 1x PBS. Samples were then washed twice with 1x PBS, once with deionised water and air-dried at room temperature in a dust free area for 2 h. Samples were then washed twice more with deionised water, left to air-dry again, followed by a final drying step using a gentle stream of nitrogen.

Samples were analysed by AFM using a Bruker multimode 8 (Bruker Corporation, Massachusetts, USA) scanning probe microscope with a Nanoscope V controller. The samples were imaged using the ScanAsyst peak force tapping mode, with RTESPA silicon cantilever probes (Bruker Corporation, Massachusetts, USA), which have a nominal spring constant of 40 N/m and a nominal tip radius of 8 nm. Data collection and processing were performed using Nanoscope software (version 1.40, Bruker Corporation). Images were scanned over a surface area of at least 10 x 10 μm at a resolution of 2048 x 2048 pixels. The scan rate was 0.2 Hz. Images were processed using the Nanoscope Analysis software (version 1.40, Bruker Corporation) and custom scripts written in Matlab to remove the sample surface tilt and scanner bow. Three dimensional (3-D) representations of the data in height and peak-force error channels were subsequently rendered in Nanoscope Analysis.

- 1. *Cryopreservation and resuscitation of infected cells*

Medium was discarded from infected cell cultures and the cell monolayer was washed once with 1x PBS. Cells were then trypsinised and suspended in 9 mL of cell culture medium prior to centrifugation at 500 *g* for 5 min at 4°C. The supernatant was discarded and the pellet suspended in 10 mL of cryo-media (RPMI-1640, 20% FBS, 10% DMSO). The cell suspension was then aliquoted into 2 mL samples in cryo-tubes and stored at -80°C inside a Mr. Frosty ^TM^ freezing container (ThermoFisher Scientific, UK, Cat No 5100-0001). The cells were left for a minimum of 2 weeks before resuscitation. To resuscitate cells, cryo-tubes were removed from the freezer and immediately thawed at 37°C in a water bath for 5 min. The cell suspension was then added to 8 mL of cell culture medium, mixed gently, and then centrifuged at 500 *g* for 5 min at 4°C. The supernatant was discarded and the cell pellet suspended in 10 mL of pre-warmed cell culture medium.

## Preparation of oocysts for MALDI-ToF MS – lipid analysis

Aliquots of 1.25 x 10^5^ or 2.5 x 10^5^ cattle-produced or COLO-680N-produced oocysts suspended in 1x PBS were pelleted at 2,100 *g* for 8 min. A matrix buffer was prepared by dissolving 20 mg/mL (saturated) of alpha-cyano-4-hydroxycinnamic acid matrix into a 40% acetonitrile 0.15% trifluoroacetic acid, which was then placed in a sonicating water bath for 15 min. Undissolved matrix was subsequently removed by centrifugation at 16,000 *g* for 10 min. The oocysts were re-suspended in 50 μL of matrix buffer. Samples were left to incubate in suspension for 30 min. The samples were then re-suspended by gently tapping the tubes before 1 μL samples were added to 384 MTP ground steel MALDI-ToF plate wells (Bruker) in triplicate. Samples were allowed to air dry before the plate was placed into the MALDI-ToF MS instrument (Bruker UltrafleXtreme).

For negative polarity compounds the following set-up was used: Laser frequency: 2000 Hz;

ion sources: (1) 19.94 kV, (2) 17.79 kV; lens 6.09 kV; reflector 1: 21.22 kV, 2: 10.74 kV; suppress at 500 Da; pulsed ion extraction 80 nS; matrix suppression mode- deflection; range 600-2000 Da; sample rate 1 Gs/s; for each sample 7500 shots were summed and saved.

For positive polarity compounds the following set up was used: laser frequency: 2000 Hz; ion sources: (1) 24.92 kV, (2) 22.27 kV; lens 7.47 kV; reflector 1: 26.53 kV, 2: 13.39 kV; suppress at 500 Da; pulsed ion extraction 80 nS; matrix suppression mode- deflection; range 600-2000 Da; sample rate 1 Gs/s; for each sample 7500 shots were summed and saved.

The MS data files were exported from the Bruker Flex Analysis software in ASCII format for pre-processing in MATLAB. Pre-processing was performed via a combination of resampling, baseline correction, application of a Savitzky-Golay filter, normalisation and automatic peak alignment as established in Povey et al. (2014). A Principle Component Analysis (PCA) model was then generated from the spectra, using the eigenvector Partial Least Squares (PLS) toolbox. The separation or merging of the groups was then used as an indicator of the differences or similarities between spectra, respectively, whilst eliminating bias. The results of the PCA produced three constituent components, PC1-PC3, which represented a summation of the differences observed between/within the spectra. A high combined PC1 and PC2 (>95%) score indicated that two dimensional (2-D) representation of the graphical data was a sufficient means of displaying the results of the PCA. Due to the nature of the data, the identity of the PCs is abstract.

## Preparation of whole cells for MALDI-ToF MS

Cell cultures were trypsinised and cell numbers adjusted to 3 x 10^4^ cells/mL. One mL of each sample was then transferred into a fresh tube and pelleted at 500 *g* for 5 min at 4 ^o^C. The samples were then mixed in the alpha cyano matrix as per the lipidomics protocol.

To analyse the cell pellets, the MALDI-ToF MS instrument (Bruker UltrafleXtreme) was calibrated before use with the commercially available Bruker Calibration Standard 1 protein mixture (Bruker, part number 206355). The spectra of the intact cell pellets were then collected using the MALDI-ToF MS instrument settings described below and the inbuilt calibration program for this calibration mixture provided with the instrument.

The spectra were collected using the following settings on the mass spectrometer: linear mode; laser frequency: 500 Hz.

A positive polarity was implemented with the following set up was used: ion sources: (1) 24.93 kV, (2) 23.08 kV; lens 7.49 kV; suppress at 5000 Da; pulsed ion extraction: 400 nS; range 5000–60,000 Da; sample rate 0.13 Gs/s; resolution enhanced 100 mV electronic gain; smooth high. For each sample 3600 shots were summed and saved.

The MS data files were exported from the Bruker Flex Analysis software in ASCII format for pre-processing, as described above. The samples were then analysed via PCA, as described above, to observe if infection state affected the protein content of the sample to a degree by which it could be detected via the lipid finger-printing technique. Due to the nature of the data, the identity of the PCs is abstract.

## Scanning electron microscopy (SEM)

Samples were prepared using the same method as with the specimens for AFM with the exception that instead of mica the sample was deposited on a 15 mm SEM carbon tape disc. The samples were visualised in a Hitachi s3400 variable pressure SEM at 50 pa and 5 kV.

- 1. *Fluorescence microscopy using C. parvum Iowa strain*

COLO-680N cells were seeded in Nunc Lab-tek 2-well chamber slides (2 ml) and infected with *Cryptosporidium parvum* (Iowa strain) with a MOI of 2. For comparison, control non-infected cells were seeded on the same day. At 48 h p.i. the supernatant was replaced by fresh media for all samples.

On Day 10 p.i,. the supernatant of infected cells (containing oocysts and COLO-680N cells and debris) was filtered through a 40 µm nylon Corning cell strainer. The flow-through was spun down at 2,000 *g* for 8 min. The supernatant was then discarded, the oocysts and debris were resuspended in 100 µl of 1x PBS and added to poly-L lysine coated slides. The slides were rinsed once in 1x PBS, then the oocysts were fixed with 4% paraformaldehyde for 30 min. The slides were rinsed once more in 1x PBS and blocked overnight in 3% filtered BSA. The slides were washed once in 1x PBS and one drop of Crypt-a-glo (Waterborne Inc.) was added for 1 h. The slide was quickly rinsed and mounting media containing DAPI (Sigma-Aldrich, Cat No F6057) were then added prior to covering with a coverslip and sealing with nail polish.

For COLO-680N cells, the chamber was washed twice with 1xPBS and the slides were prepared as described above, except that after fixation, the cells were treated for 10 min with 0.1% Triton in 1X PBS to permeabilise cells. COLO-680N were stained with either Crypt-a-glo or Sporo-glo (Waterborne Inc).

**References**

Alcantara Warren, C., Destura, R.V., Sevilleja, J.E., Barroso, L.F., Carvalho, H., Barrett, L.J., O'Brien, A.D., and Guerrant, R.L., 2008. Detection of epithelial-cell injury, and quantification of infection, in the HCT-8 organoid model of cryptosporidiosis. J. Infect. Dis. 198, 143-149.

Bhalchandra, S., Ludington, J., Coppens, I., and Ward, H.D., 2013. Identification and characterization of *Cryptosporidium parvum* clec, a novel C-type lectin domain-containing mucin-like glycoprotein. Infect. Immun. 81, 3356-3365.

Bouzid, M., Hunter, P.R., McDonald, V., Elwin, K., Chalmers, R.M., Tyler, K.M., 2013. A new heterogeneous family of telomerically encoded *Cryptosporidium* proteins. Evol. Appl. 6, 207-217.

Bustin, S.A., Benes, V., Garson, J.A., Hellemans, J., Huggett, J., Kubista, M., Mueller, R., Nolan, T., Pfaffl, M.W., Shipley, G.L., Vandesompele, J., Wittwer, C.T., 2011. Primer sequence disclosure: A clarification of the MIQE guidelines. Clin. Chem. 57, 919-921.

Chen, F., Huang, K.H., 2006. Study on methods for isolation and purification of *Cryptosporidium parvum* oocysts from mouse feces. Zhongguo Ji Sheng Chong Xue Yu Ji Sheng Chong Bing Za Zhi 24, 219-222.

Feng, H., Nie, W., Bonilla, R., Widmer, G., Sheoran, A., Tzipori, S., 2006. Quantitative tracking of *Cryptosporidium* infection in cell culture with CFSE. J. Parasitol. 92, 1350-1354.

Girouard, D., Gallant, J., Akiyoshi, D.E., Nunnari, J., Tzipori, S., 2006. Failure to propagate *Cryptosporidium* spp. in cell-free culture. J. Parasitol. 92, 399-400.

Sharling, L., Liu, X., Gollapalli, D.R., Maurya, S.K., Hedstrom, L., Striepen, B., 2010. A screening pipeline for antiparasitic agents targeting *Cryptosporidium* inosine monophosphate dehydrogenase. PLoS Negl Trop. Dis. 4, e794.

Upton, S.J., Tilley, M., Nesterenko, M.V., Brillhart, D.B., 1994. A simple and reliable method of producing in vitro infections of *Cryptosporidium parvum* (apicomplexa). FEMS Microbiol. Lett. 118, 45-49.

Wu, Z., Nagano, I., Matsuo, A., Uga, S., Kimata, I., Iseki, M., Takahashi, Y., 2000. Specific PCR primers for *Cryptosporidium parvum* with extra high sensitivity. Mol. Cell. Probes 14, 33-39.
