# Supplementary material for: A cell culture platform for Cryptosporidium that enables long-term cultivation and new tools for the systematic investigation of its biology
Source: Int J Parasitol. 2018 Mar;48(3-4):197–201. doi: 10.1016/j.ijpara.2017.10.001 (PMC5854368; doi:10.1016/j.ijpara.2017.10.001)
Supplement: Supplementary Figs. S1–S10 [file mmc2.pdf]

Supplementary Figure S1

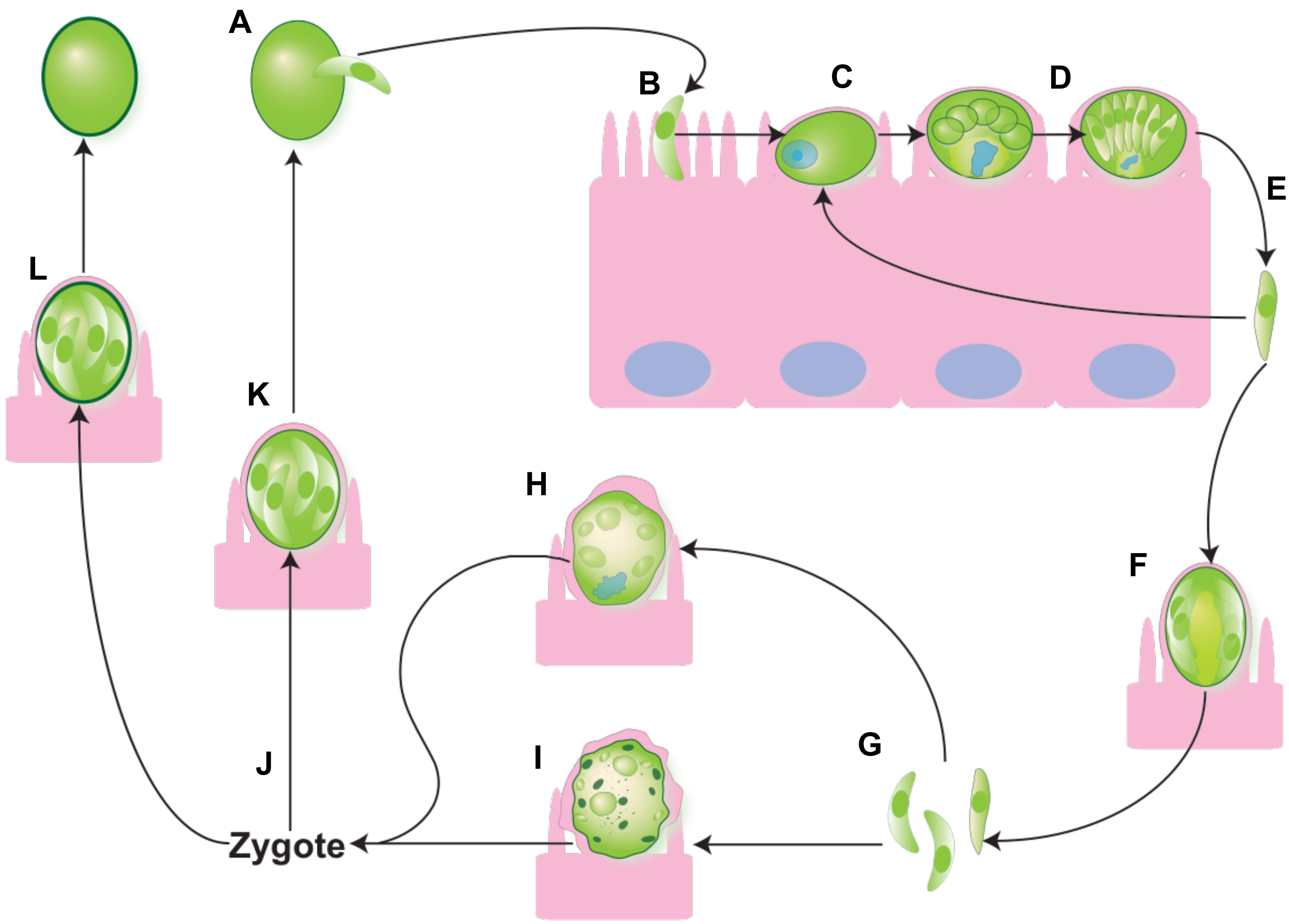

Supplementary Figure S2

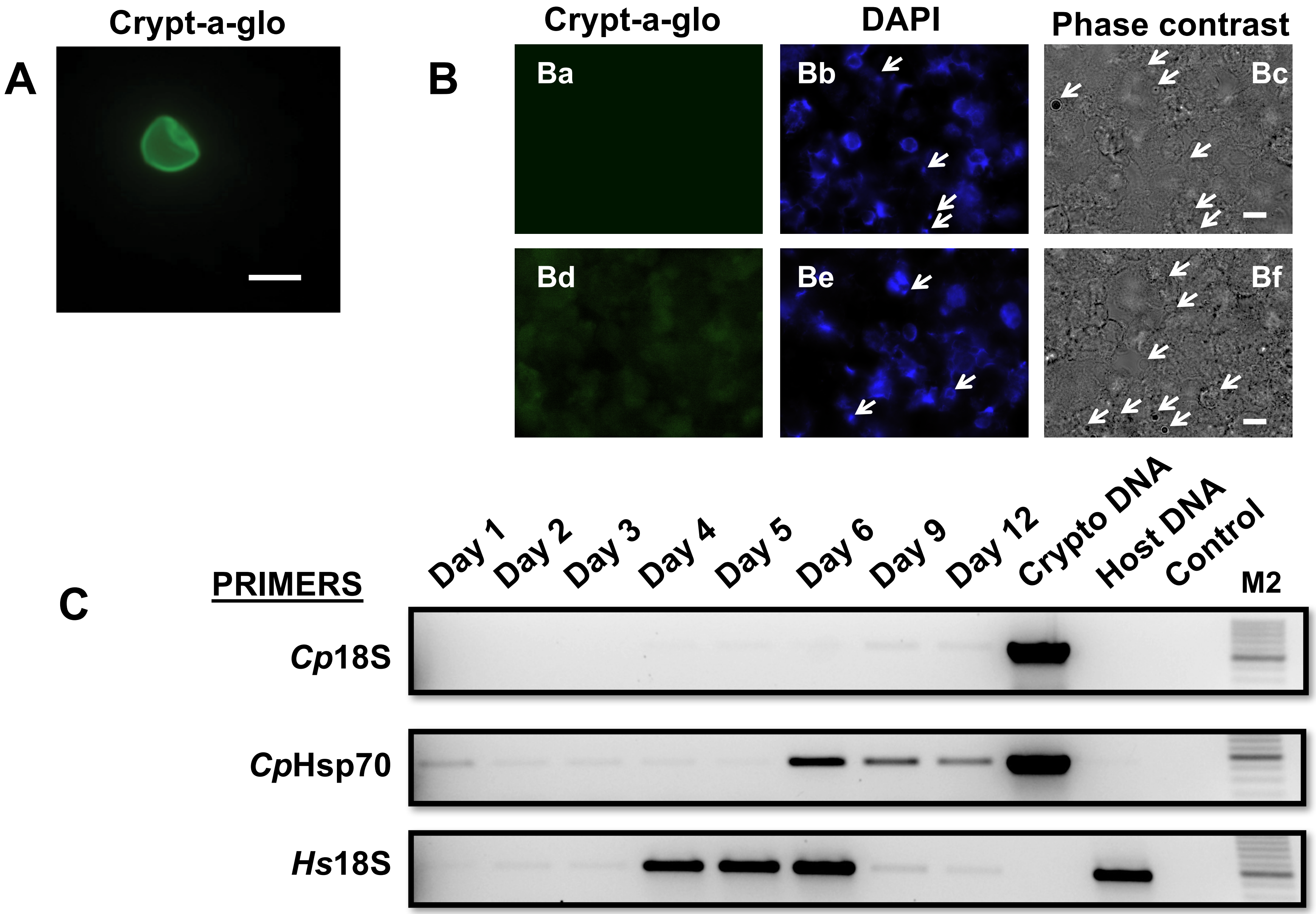

Supplementary Figure S3

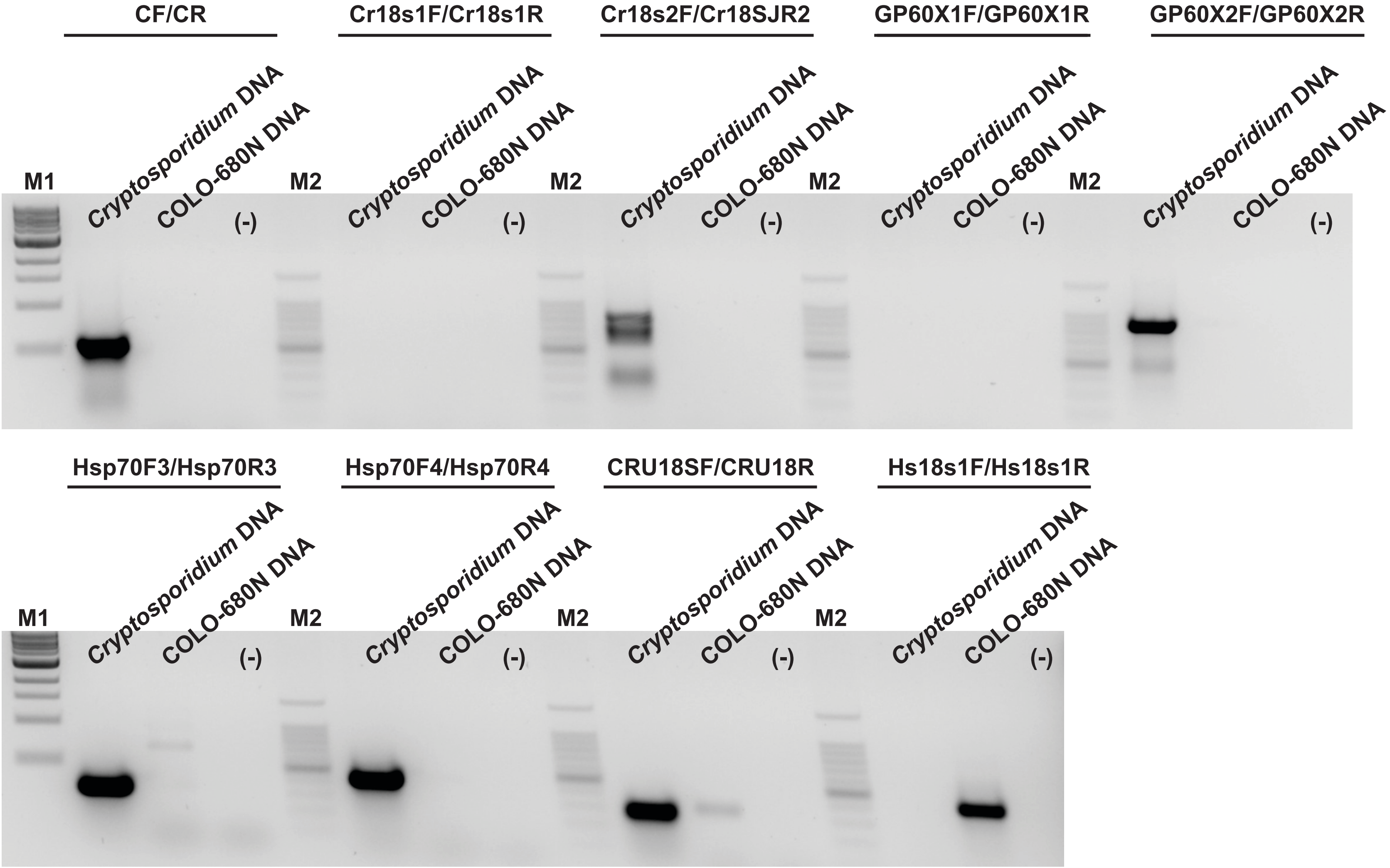

Supplementary Figure S4

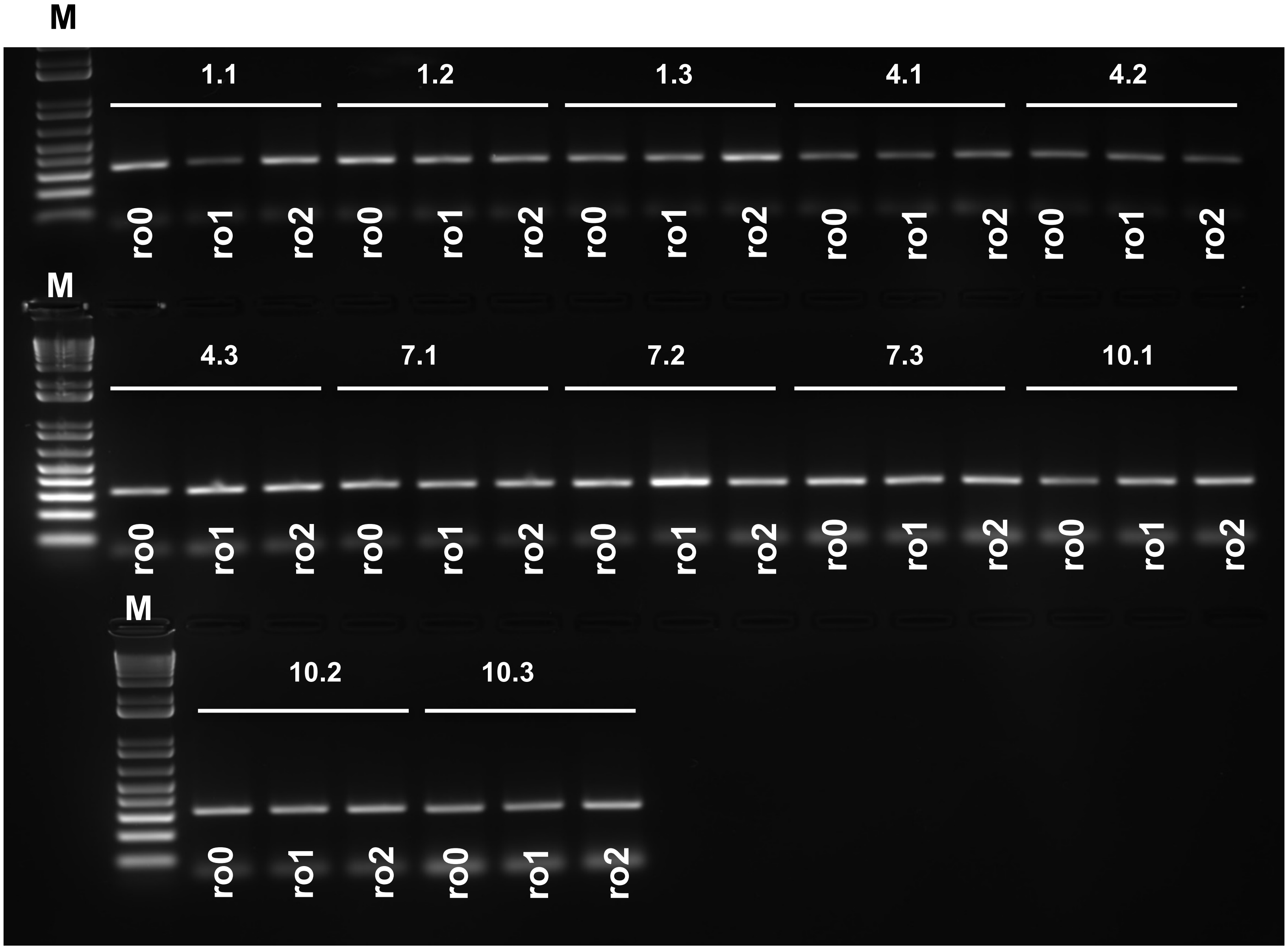

Supplementary Figure S5

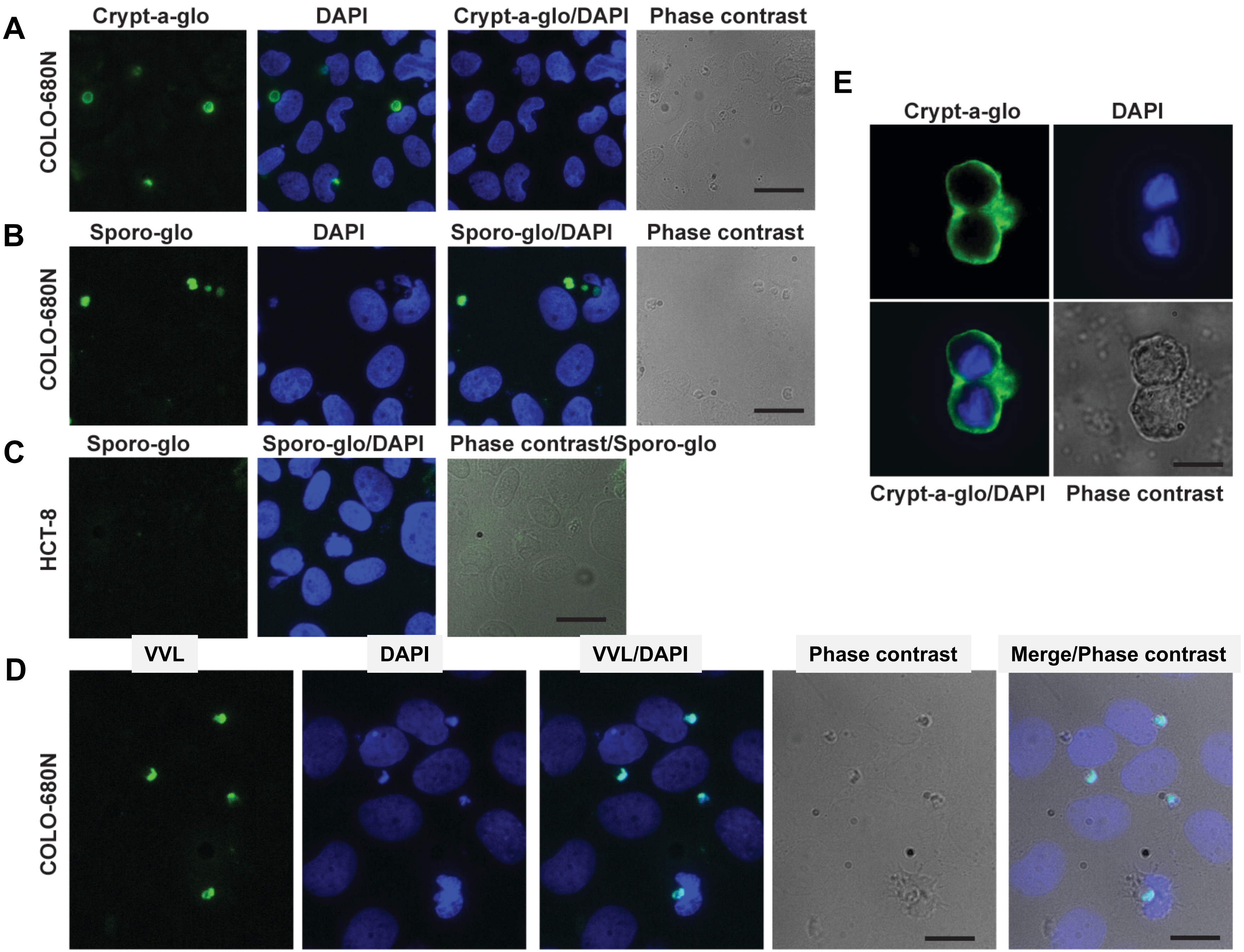

Supplementary Figure S6

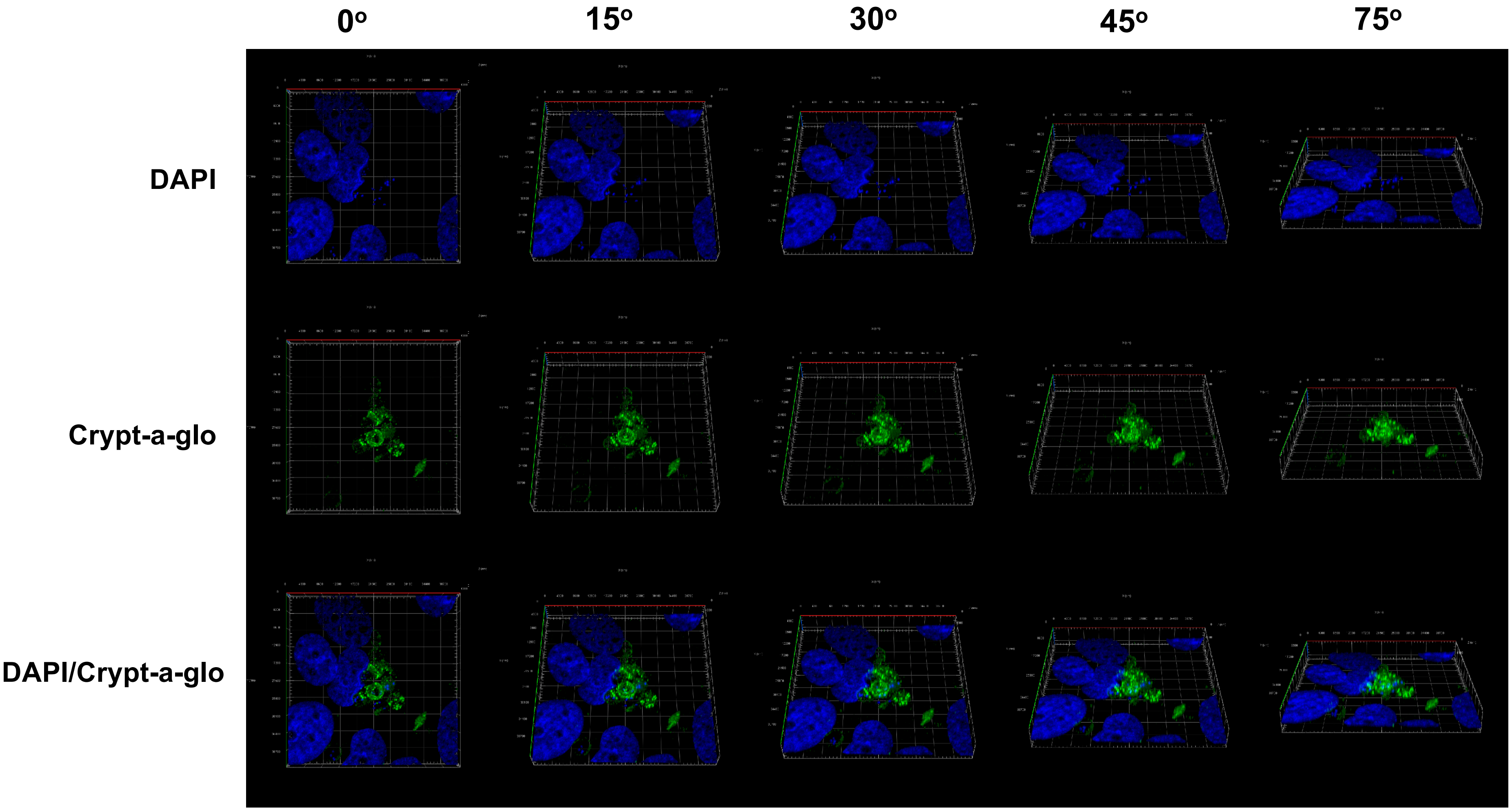

Supplementary Figure S7

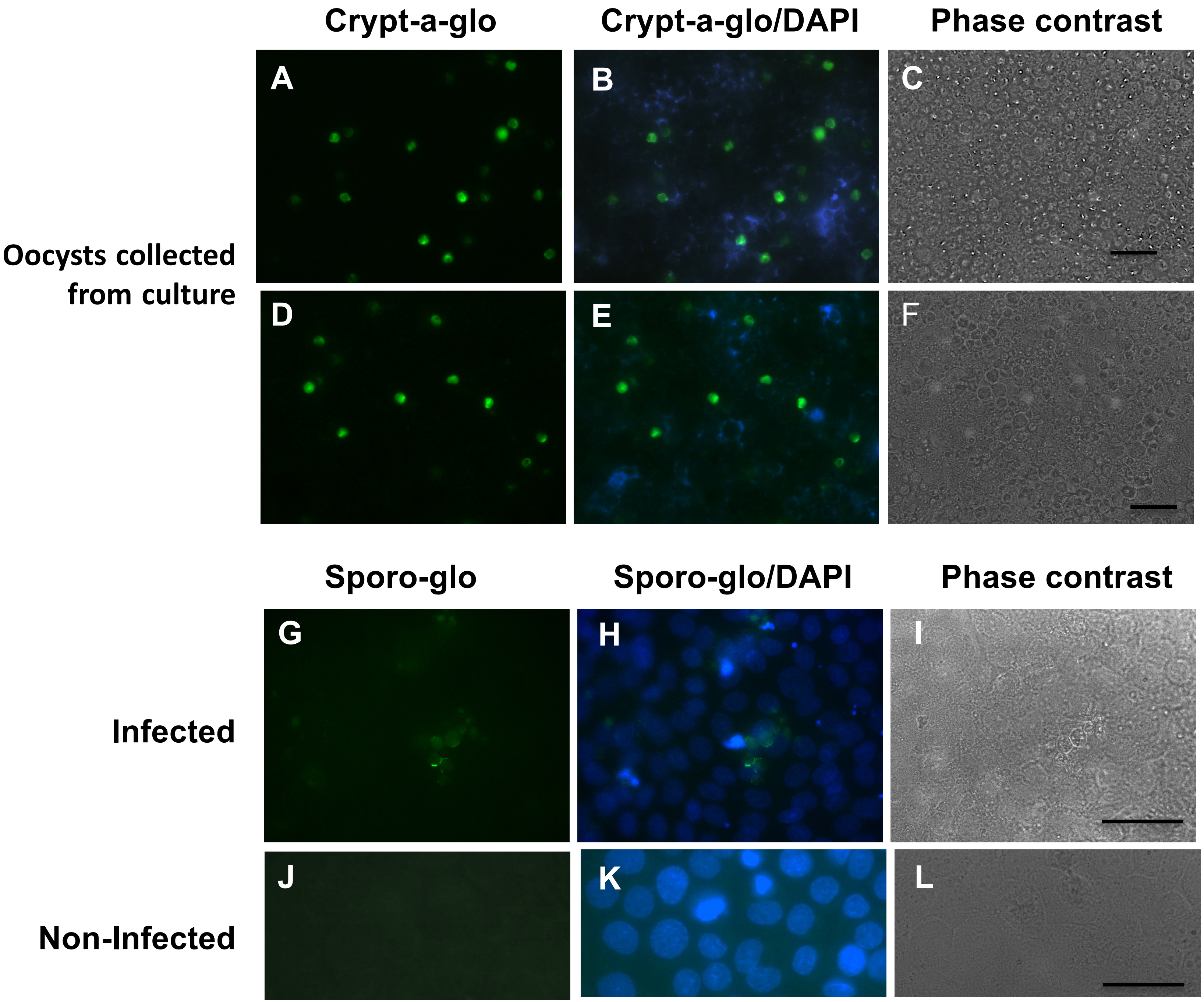

Supplementary Figure S8

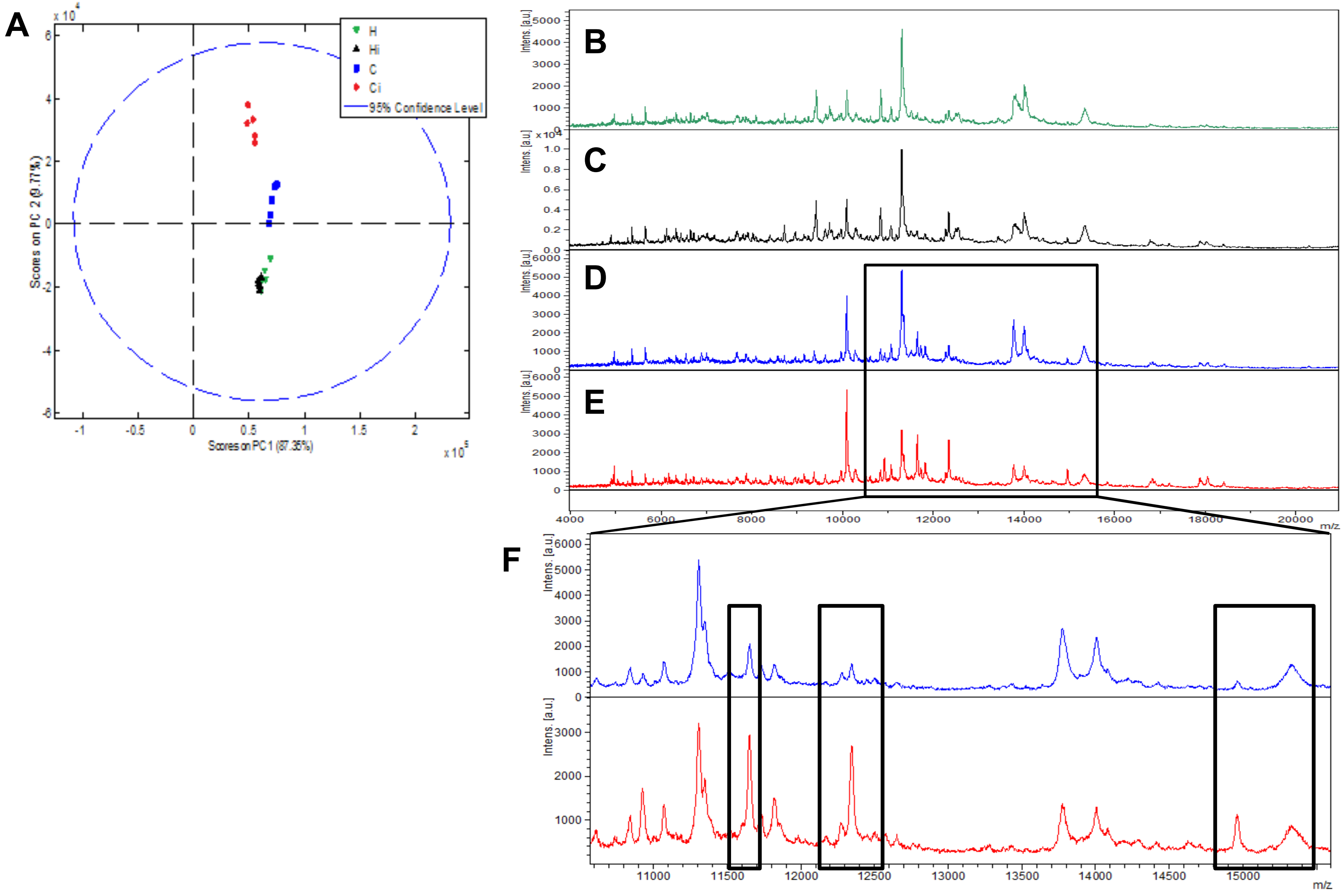

Supplementary Figure S9

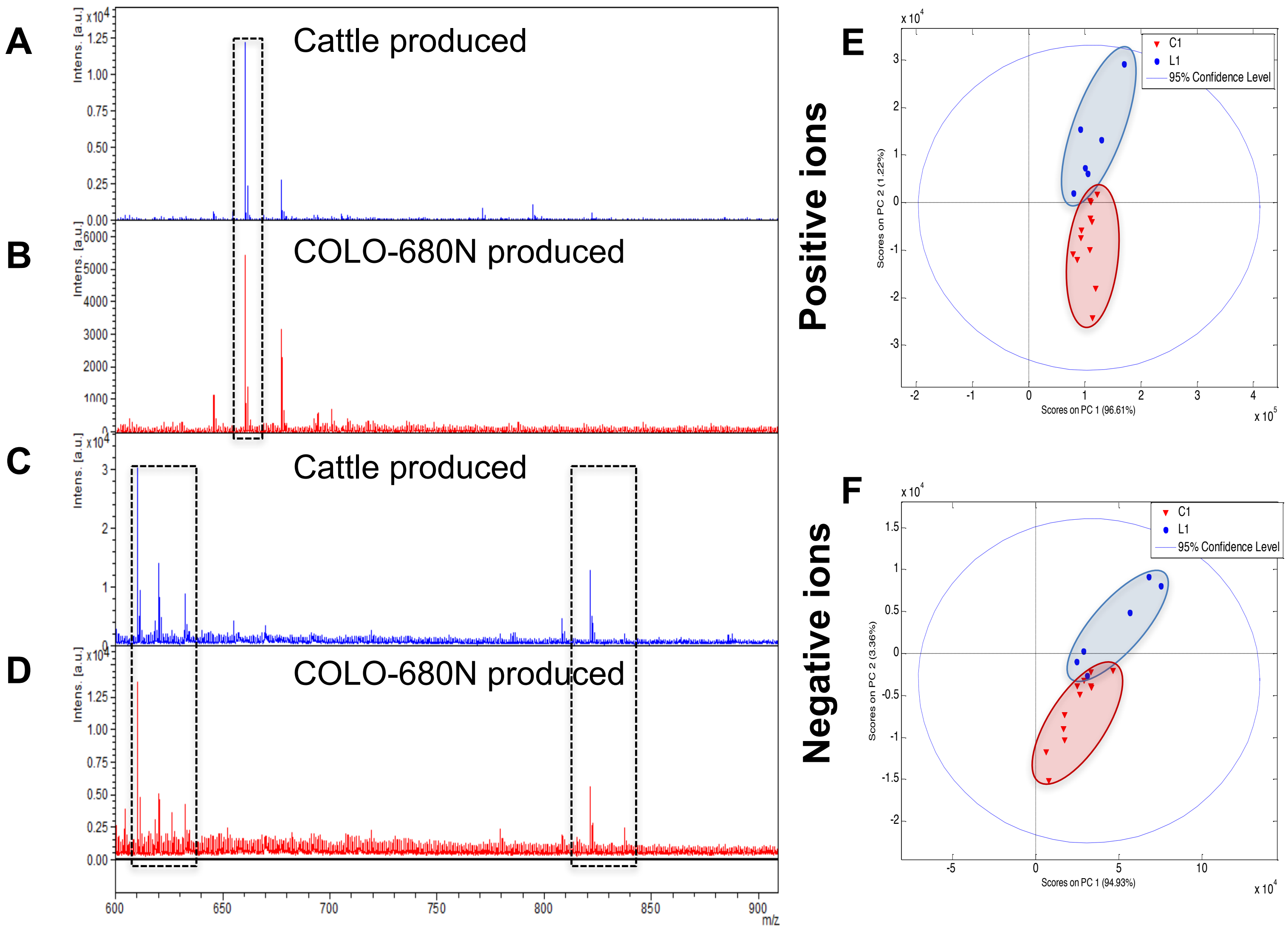

Supplementary Figure S10

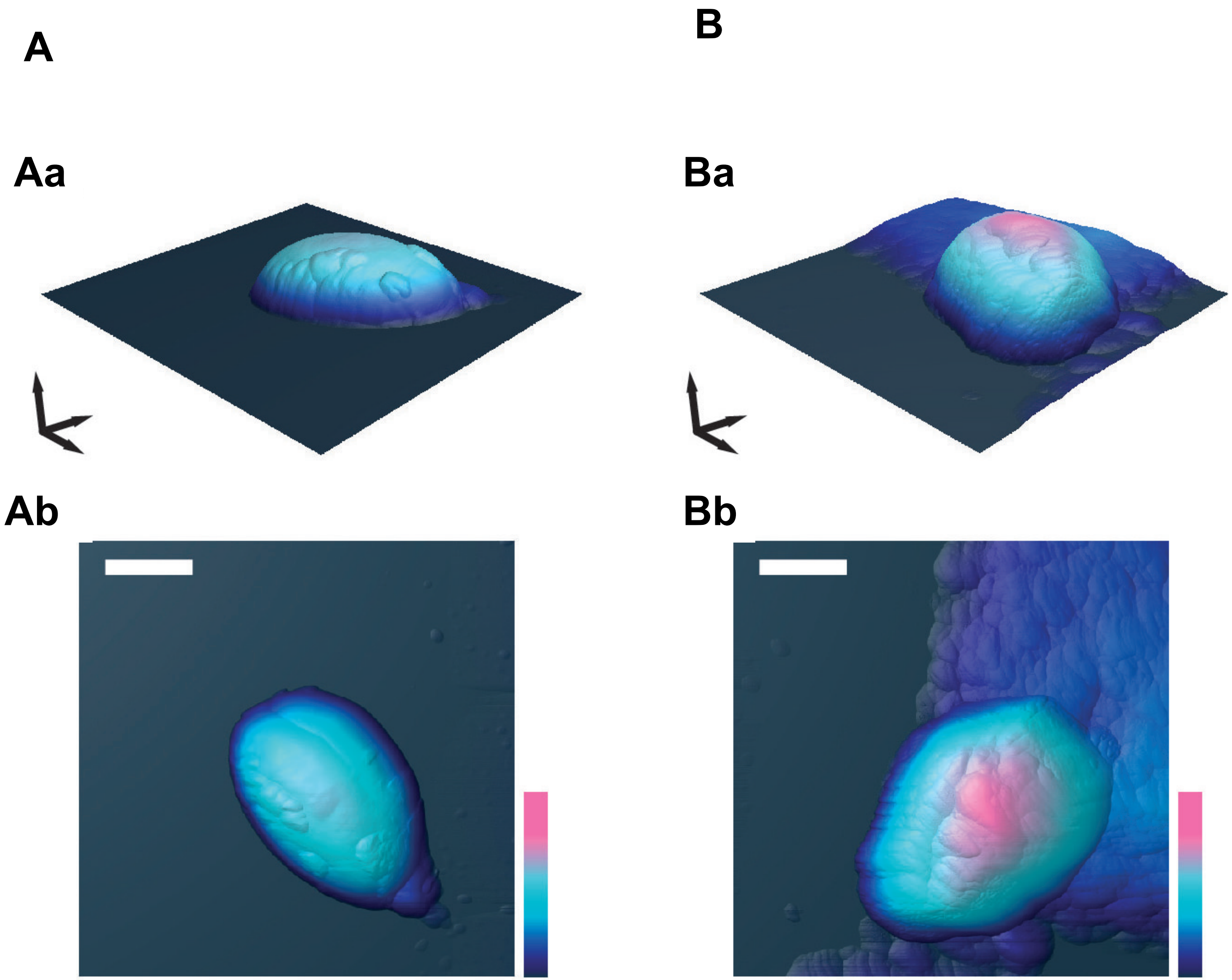

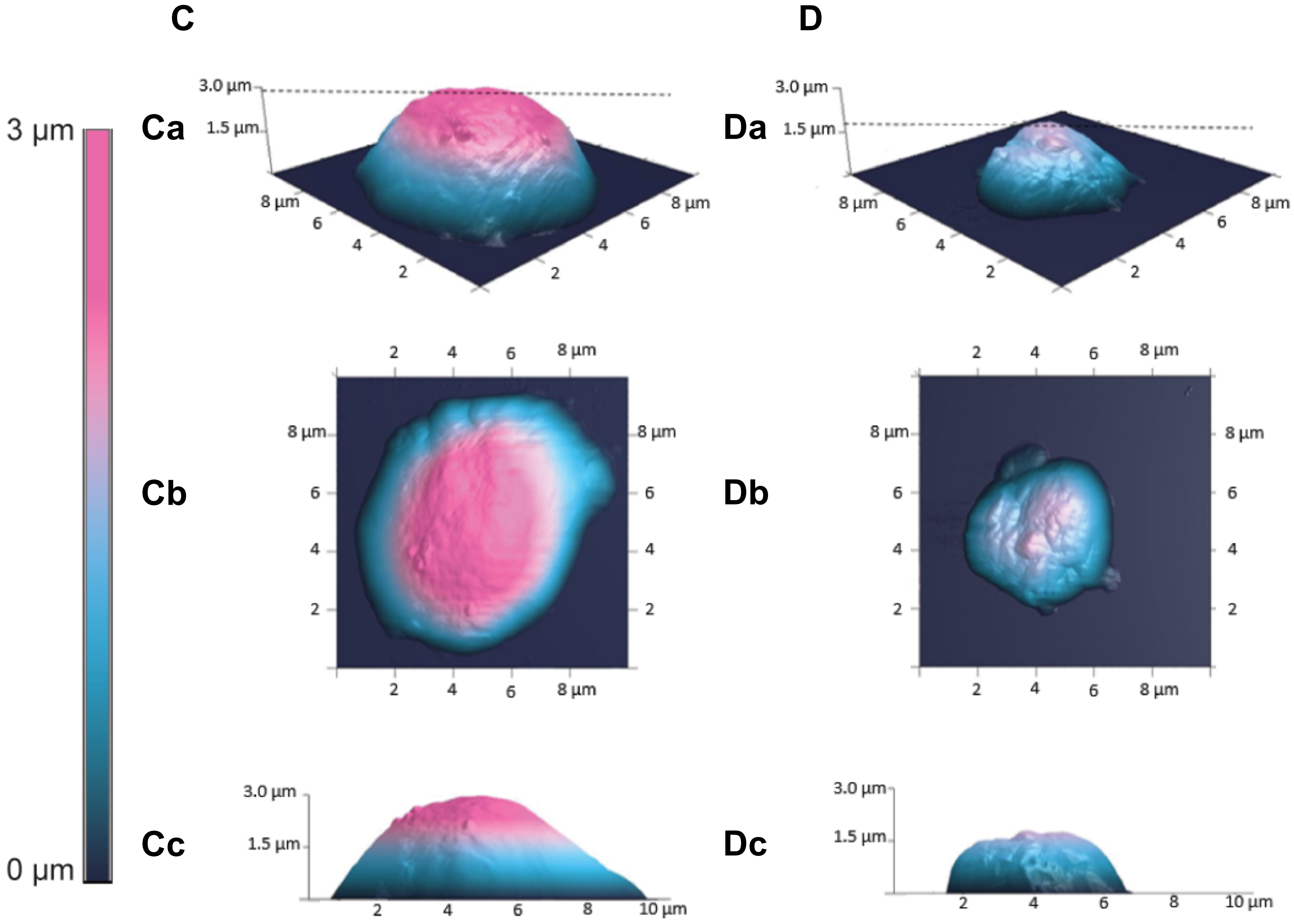

C
D
3 µm
0 µm
Ca
Cb
Cc
Da
Db
Dc
3.0 µm
1.5 µm
8 µm
6
4
2
10 µm
