# Supplementary material for: A cell culture platform for Cryptosporidium that enables long-term cultivation and new tools for the systematic investigation of its biology
Source: Int J Parasitol. 2018 Mar;48(3-4):197–201. doi: 10.1016/j.ijpara.2017.10.001 (PMC5854368; doi:10.1016/j.ijpara.2017.10.001)
Supplement: Supplementary Table S1 [file mmc3.docx]

**Supplementary Table S1.** Observations of *Cryptosporidium* infection and propagation in various cell lines.

| Cell type | Location | Notes | Oocysts/ml of culture, after 2 weeks incubation post infection with 1 x 10^5^ | Decision |
| --- | --- | --- | --- | --- |
| COLO-680N | Oesophagus from human | Many high contrast, retractile, circular bodies seen suspended in the media, with a myriad of different internal structures witnessed. This cell type displays a prevalence to naturally form circular 'holes' between cells, which made identifying intracellular stages hard initially. | **3.75 x 10^6^** | Follow-up |
| DLD-1 PAR | Human colon adenocarcinoma | Cell displayed a large amount of detachment, suggesting stress. Some swelling was observed compared to the controls, suggesting the presence of parasitophorous vacuoles. | **4.7 x 10^4^** | Return too low |
| HCT-8 | Colorectal adenocarcinoma | High contrast 'retractile' bodies of a circular shape were observed in some cultures. Infected cultures displayed a 'swollen' physiology compared to the controls. | **2.5 x 10^5^** | Control |
| HCT-15 | Colorectal adenocarcinoma | High contrast 'retractile' bodies of a circular shape were observed in some cultures. Infected cultures displayed a 'swollen' physiology compared to the controls | **2.5 x 10^5^** | Return too low |
| KYSE | Oesophagus from human | Whilst no life cycles stages aside from the oocyst could be detected, the culture displayed a large amount of cell detachment, suggesting stress. Most likely the oocysts were a remnant of the original infection. | **1.25 x 10^5^** | Return too low |
| MKN-1 | Gastric carcinoma | Cells from the infected culture did not display any characteristics different from the controls, with no evidence of infection. | **3.4 x 10^5^** | Return too low |
| SJSA-1 | Osteosarcoma; multipotential sarcoma | Swelling observed and the presence of motile forms suggested the existence of a viable infection. | **3.1 x 10^5^** | Return too low |

Shading indicates the successful cell line that was followed up for further studies.
