# Supplementary material for: A cell culture platform for Cryptosporidium that enables long-term cultivation and new tools for the systematic investigation of its biology
Source: Int J Parasitol. 2018 Mar;48(3-4):197–201. doi: 10.1016/j.ijpara.2017.10.001 (PMC5854368; doi:10.1016/j.ijpara.2017.10.001)
Supplement: Supplementary Table S2 [file mmc4.docx]

**Supplementary Table S2.** *Cryptosporidium-*specific primers used during this project.

| Primer region | Primer name | Primer sequence |
| --- | --- | --- |
| *Cryptosporidium* COWP primers | CF (Cry-15) | GTAGATAATGGAAGAGATTGTG |
|  | CR (Cry-9) | GGACTGAAATACAGGCATTATCTTG |
| *Cryptosporidium* 18s primers | Cp18s1F | TTCTAGAGCTAATACATGCG |
|  | Cp18s1R | CCCATTTCCTTCGAAACAGGA |
|  | Cp18s2F | GGAAGGGTTGTATTTATTAGATAAAG |
|  | Cp18SJR2 | CTCATAAGGTGCTGAAGGAGTA |
| *Cryptosporidium* GP60 primers | GP60X1F (AL3531) | ATAGTCTCCGCTGTATTC |
|  | GP60X1R (AL3535) | GGAAGGAACGATGTATCT |
|  | GP60X2F (AL3532) | TCCGCTGTATTCTCAGCC |
|  | GP60X2R (AL3534) | GCAGAGGAACCAGCATC |
| *Cryptosporidium* HSP70 primers | CpHsp70F3 (2°F) | GCTGSTGATACTCACTTGGGTGG |
|  | CpHsp70R3 (2°R | CTCTTGTCCATACCAGCATCC |
|  | CpHsp70F4 (1°F) | GGTGGTGGTACTTTTGATGTATC |
|  | CpHsp70R4 (1°R) | GCCTGAACCTTTGGAATACG |
|  | CpHsp70FLJ | GCTGGTGATACTCACTTGGGTGGTG |
|  | CpHsp70RLJ | CTCTTGTCCATACCAGCATCCTTG |
| Random amplified polymorphic DNA | SB012F | ataaacaagcaggaaaaaaggt |
|  | SB012R | cgcacaagttacaaggattatt |
| *Cryptosporidium* CRU 18S primers | CRU18SF | GAGGTAGTGACAAGAAATAACAATACAGG |
|  | CRU18R | CTGCTTTAAGCACTCTAATTTTCTCAAAG |
