# Supplementary figures and images for: A cell culture platform for Cryptosporidium that enables long-term cultivation and new tools for the systematic investigation of its biology
Source: Int J Parasitol. 2018 Mar;48(3-4):197–201. doi: 10.1016/j.ijpara.2017.10.001 (PMC5854368; doi:10.1016/j.ijpara.2017.10.001)

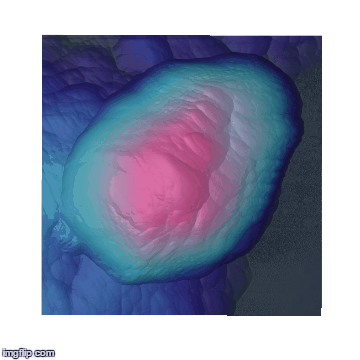

Supplement: Supplementary Movie S1 — Cryptosporidium parvum oocyst captured by Atomic Force Microscopy (AFM). Three-dimensional topology animation displaying the overall morphology of the COLO-680N-produced oocyst displayed in Supplementary Fig. S10A. [file mmc5.gif]
